# Capacity of Human Dental Follicle Cells to Differentiate into Neural Cells* In Vitro*

**DOI:** 10.1155/2017/8371326

**Published:** 2017-02-05

**Authors:** Shingo Kanao, Naomi Ogura, Kosuke Takahashi, Ko Ito, Masaaki Suemitsu, Kayo Kuyama, Toshirou Kondoh

**Affiliations:** ^1^Department of Maxillofacial Surgery, Nihon University School of Dentistry at Matsudo, Matsudo, Japan; ^2^Research Institute of Oral Science, Nihon University School of Dentistry at Matsudo, Matsudo, Japan; ^3^Department of Oral Pathology, Nihon University School of Dentistry at Matsudo, Matsudo, Japan

## Abstract

The dental follicle is an ectomesenchymal tissue surrounding the developing tooth germ. Human dental follicle cells (hDFCs) have the capacity to commit to differentiation into multiple cell types. Here we investigated the capacity of hDFCs to differentiate into neural cells and the efficiency of a two-step strategy involving floating neurosphere-like bodies for neural differentiation. Undifferentiated hDFCs showed a spindle-like morphology and were positive for neural markers such as nestin, *β*-III-tubulin, and S100*β*. The cellular morphology of several cells was neuronal-like including branched dendrite-like processes and neurites. Next, hDFCs were used for neurosphere formation in serum-free medium containing basic fibroblast growth factor, epidermal growth factor, and B27 supplement. The number of cells with neuronal-like morphology and that were strongly positive for neural markers increased with sphere formation. Gene expression of neural markers also increased in hDFCs with sphere formation. Next, gene expression of neural markers was examined in hDFCs during neuronal differentiation after sphere formation. Expression of* Musashi-1* and* Musashi-2*,* MAP2*,* GFAP*,* MBP*, and* SOX10* was upregulated in hDFCs undergoing neuronal differentiation via neurospheres, whereas expression of* nestin* and *β-III-tubulin* was downregulated. In conclusion, hDFCs may be another optimal source of neural/glial cells for cell-based therapies to treat neurological diseases.

## 1. Introduction

Neurodegenerative disorders are characterized by the loss or atrophy of neurons, leading to functional impairment. Various approaches have been proposed to have a beneficial effect on peripheral nerve regeneration, including application of an electric field, administration of neurotrophic factors, and transplantation of stem cells [1–4]. Implantation of embryonic stem cells, neural stem cells and mesenchymal stem cells (MSCs) is beneficial for peripheral nerve regeneration. MSCs are multipotent stem cells that are capable of differentiating into multiple cell types [5, 6]. The* in vitro* growth of undifferentiated MSCs, followed by induction of neural cell differentiation and subsequent transplantation, is an important modality for cell therapy to treat neurodegenerative disease [7, 8]. Although human bone marrow is generally used as the major source of MSCs to treat neurodegenerative disease [9, 10], MSCs can be derived from all postnatal tissues.

The dental follicle is an ectomesenchymal tissue derived from the neural crest and surrounds the tooth germ. The dental follicle contains stem cells and/or progenitor cells of the periodontium. Human dental follicle cells (hDFCs) have the capacity to commit to differentiation into multiple cell lineages such as osteoblastic, adipogenic, and neurogenic lineages [11–13]. hDFCs are a major source of stem cells in adults, as they can be easily obtained during various surgical procedures, such as the extraction of impacted teeth. hDFCs therefore have great potential for regenerative purposes in cell therapy. Our group previously compared the gene expression profiles between hDFCs and MSCs from human bone marrow (hMSCs) to investigate whether hDFCs are a useful cell source for applications in clinical tissue regeneration. The expression of MSC markers and growth factor receptors was similar in hDFCs and hMSCs, whereas the expression pattern of homeobox genes differed between the two cell types. We suggested that hDFCs may have the capacity to differentiate into neural cells because hDFCs express markers for neural stem cells such as* nestin* and* notch-1*. However, few studies have examined the potential for neuronal-like cell differentiation by hDFCs compared to that for osteoblast differentiation.

The aim of this study has been to investigate the capacity of hDFCs to differentiate into neural stem cells for nervous regeneration in nervous disease and injuries. We verified the positive expression of neuronal markers in response to induction using immunocytochemistry and real-time PCR. We also investigated the efficiency of a two-step strategy involving the generation of floating neurosphere-like bodies for neural differentiation by hDFCs.

## 2. Materials and Methods

### 2.1. Isolation and Culture of hDFCs

hDFCs were obtained using a previously reported method [11]. The use of hDFCs was approved by the Ethics Committee of Nihon University School of Dentistry at Matsudo (Recognition number: EC 15-10-036-1 and EC 15-040). Briefly, normal human impacted third molars were surgically removed and collected from two patients (one male and one female: 14 years of age) who gave informed consent. Dental follicle tissues were washed in phosphate-buffered saline (PBS), minced with sterilized scalpels, and digested in a solution of 0.1 U/ml collagenase type I and 1 U/ml dispase (Roche, Basel, Switzerland) for 1 h at 37°C. hDFCs attached to 100-mm culture plates and were grown in MSC growth medium (GM; consisting of MSC basal medium supplemented with fetal bovine serum, l-glutamine, and penicillin/streptomycin; Lonza, Basel, Switzerland) in a humidified incubator (CO_2_ incubator MCO-175M; Panasonic, Tokyo, Japan) in 5% CO_2_ in air at 37°C. hDFCs from the 5th to 6th passage were used for the following experiments.

### 2.2. Neuronal Differentiation

Two different protocols were used for* in vitro* differentiation into neuronal-like cells from hDFCs.

*One-Step Method.* hDFCs were seeded at 4.0 × 10^4^ cells/dish on 35-mm dishes coated with fibronectin (BioCoat™, Corning, Corning, NY) in GM in a humidified incubator in 5% CO_2_ in air at 37°C. After the cells became 50–70% confluent, medium was replaced with MSC Neurogenic Differentiation Medium (NDM; Promocell, Heidelberg, Germany). hDFCs were cultured for 7 days, and medium was replaced every 2 days.

*Two-Step Method.* The two-step method involved the generation of floating neurosphere-like bodies [14, 15]. hDFCs were plated on 96-well low-attachment culture plates (Hydrocell; CellSeed, Tokyo, Japan) at a density of 1.6 × 10^3^ cells/well in DMEM (Wako, Tokyo, Japan) containing B27 supplement (Thermo Fisher Scientific, Waltham, MA), 20 ng/ml epidermal growth factor (EGF; Higeta Shoyu, Tokyo, Japan), and 20 ng/ml fibroblast growth factor 2 (bFGF; PeproTech, Rocky Hill, NJ). After 48 h, the cells were transferred to a fibronectin-coated dish. After 24 h, the medium was replaced with neuronal differentiation medium, which was replaced every 2 days.

### 2.3. Immunocytochemistry

Cells were fixed with 10% formalin neutral buffer solution for 30 min at room temperature, permeabilized in 0.1% Triton X-100, and blocked with 10% normal goat serum (Thermo Fisher Scientific) in PBS. Primary antibodies were applied for 1 h at room temperature, cultures were washed, and then secondary antibodies were incubated for 1 h at room temperature in the dark. The following antibodies and final dilutions were used: primary antibodies: mouse anti-nestin (ab22035, 1 : 200; abcam, Cambridge, UK); mouse anti-*β*-tubulin III (ab7751, 1 : 500; abcam); rabbit anti-S100*β* (ab52642, 1 : 250; abcam); secondary antibodies: goat anti-mouse, anti-human conjugated to Alexa Flour® 488 (A-11001, Thermo Fisher Scientific). Nuclei were counterstained with 4,6-diamidio-2 phenylindole (ProLong® Gold Antifade Mountant with DAPI; Thermo Fisher Scientific).

### 2.4. Imaging and Image Processing

Bright-field images of neuronal differentiation cultures were acquired using an Olympus CKX41 fitted with a DP20 (Olympus, Tokyo, Japan). Images of neuronal differentiation cultures that were stained were acquired using an Olympus BX51 microscope equipped with a DP72 (Olympus). All digital images were processed (merge, black balance) using GIMP Portable 2.8. (GIMP Development Team.)

### 2.5. Total RNA Isolation

Total RNA was isolated using miRNeasy Mini Kits (QIAGEN, Hilden, Germany) according to the manufacturer's instructions.

### 2.6. Real-Time PCR

Complementary DNA (cDNA) was synthesized by using a GeneAmp RNA PCR Kit (Thermo Fisher Scientific). Real-time PCR was performed using a DyNAmo SYBR Green qPCR Kit (Thermo Fisher Scientific). The PCR mixture, containing 20 pmol forward and reverse primers and 2 *μ*l cDNA, was subjected to amplification with a DNA Engine Opticon 1 (Bio-Rad, Hercules, CA), with preheating at 95°C for 15 min, followed by 40 cycles of 94°C for 15 sec, 60°C or 55°C for 30 sec, and 72°C for 30 sec. Primer sequences and annealing temperatures used for real-time PCR analysis are shown in Table 1. Gene expression levels were calculated using the ΔΔC_T_ method with normalization to GAPDH [16].

### 2.7. Statistical Analysis

Data are shown as mean values ± SD. The Student's* t*-test was used for the analysis of differences.

## 3. Results

### 3.1. Neuronal Differentiation Potential of hDFCs

**Figure 1:**
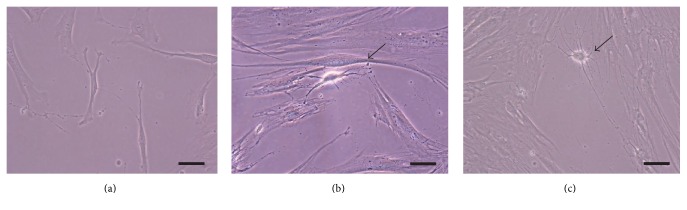
Morphologic findings of hDFCs during one-step neuronal differentiation. (a) Undifferentiated hDFCs (day 0). (b) hDFCs on day 3 after neuronal induction. (c) hDFCs on day 7 after neuronal induction. Scale bars = 50 *μ*m. Arrows indicate neural stem cell-like cells.

First, we examined the potential of hDFCs to differentiate into neuronal-like cells when transferred to appropriate conditions for neuronal differentiation.  Figure 1 shows the morphology characteristics of hDFCs cultured on a fibronectin-coated dish in NDM for 0, 3, and 7 days. Undifferentiated hDFCs attached to the plastic surface and exhibited a fibroblast-like spindle-shaped morphology (culture day 0). After 3 days of incubation in neuronal differentiation conditions, a change in cellular morphology was observed in several cells along with branched dendrites and refractile cell bodies with neurite-like processes terminating in structures resembling growth cones (arrow). Furthermore, several cells changed their morphology and became neuronal-like cells with long neurites. These projections extended further and became longer (arrow).

**Figure 2:**
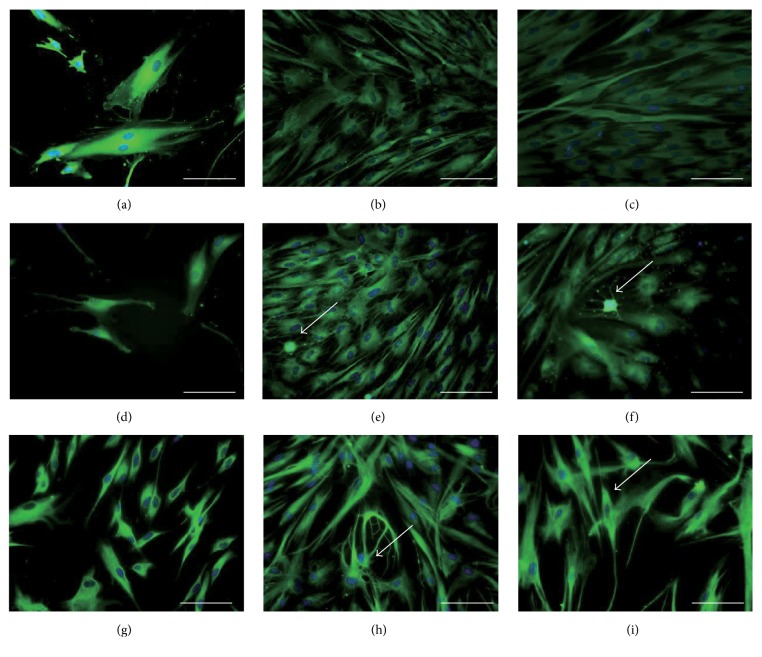
Immunofluorescence staining of neural markers in hDFCs during one-step neuronal induction. (a–c) Nestin staining. (d–f) *β*-III-Tubulin staining. (g–i) S100*β* staining. (a, d, g) Undifferentiated hDFCs (day 0). (b, e, h) hDFCs on day 3 after neuronal induction. (c, f, i) hDFCs on day 7 after neuronal induction. Scale bars = 50 *μ*m. Arrows indicate neural-like cells with branched dendrites and refractile cell bodies.

Neuronal-specific markers were examined using immunocytochemical staining. Undifferentiated hDFCs retained the expression of nestin, *β*-III-tubulin, and S100*β* (Figure 2). Staining for the neural stem cell marker nestin [17, 18] decreased following neuronal induction (Figures 2(a), 2(b), and 2(c)). Cells with a neural morphology were strongly positive for the neuronal cell marker *β*-III-tubulin after 3 and 7 days of neuronal differentiation (Figures 2(e) and 2(f)). Strong staining for the glial cell marker S100*β* [19] was observed in cells with neural morphology compared to cells with the undifferentiated phenotype on culture days 3 and 7 (Figures 2(h) and 2(i)). Furthermore, bipolar neuron-like cells and other neuron-like cells were observed on culture days 3 and 7.

**Figure 3:**
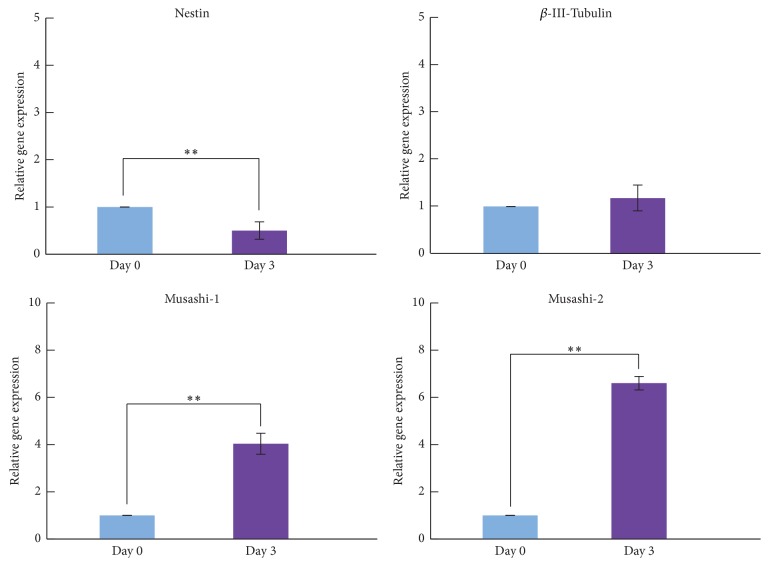
Gene expression of neural markers in hDFCs during one-step neuronal induction. Gene expression was compared in hDFCs between the undifferentiated condition and 3 days after one-step neuronal induction. Values represent means ± SD (*n* = 3). ^*∗∗*^*p* < 0.01.

To confirm further neural differentiation of hDFCs, we analyzed gene expression of the neural marker genes,* nestin*,  *β-III-tubulin*, and* Musashi-1* and* Musashi-2*.* Nestin* was downregulated in hDFCs following neuronal induction, whereas expression of  *β-III-tubulin* did not change significantly (Figure 3). Expression of the neural progenitor cell markers* Musashi-1* and* Musashi-2* [18–20] was significantly upregulated in hDFCs (Figure 3).

### 3.2. Neurosphere Formation

**Figure 4:**
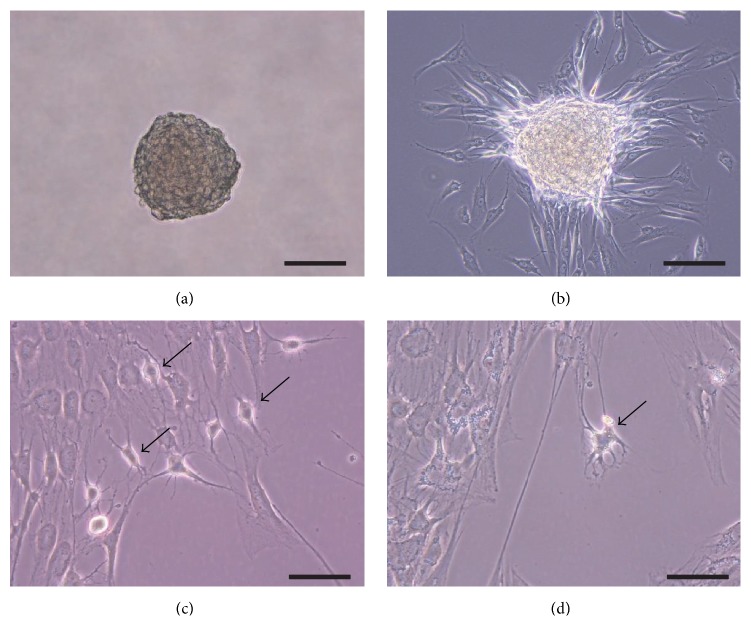
Neurosphere generation of hDFCs. (a) A floating cell sphere of hDFCs cultured in serum-free medium with FGF, EGF, and B27 supplement for 3 days. (b) The cells spread out from the neurosphere that adhered to the fibronectin-coated dish on day 0 of culture in NDM. (c) The cells extended from the neurosphere on day 3 of culture in NDM. (d) The cells extended from the neurosphere on day 7 of culture in NDM. Scale bars (a, b) = 100 *μ*m and (c, d) = 50 *μ*m. Arrows indicate neural-like cells with branched dendrites and refractile cell bodies.

The two-step strategy through neurosphere formation appeared to be more efficient for neuronal differentiation. Thus, we examined the neuronal differentiation potential of hDFCs using a protocol that included neurosphere formation in serum-free medium containing bFGF and EGF for 3 days. The cell spheres of hDFCs that formed were transferred to fibronectin-coated culture dishes, and then spindle-shaped cells spread out from the sphere after 24 h (Figure 4(b)). After 3 days of culture in NDM, the cell morphology of hDFCs changed from spindle-shaped to a neuronal-like cell phenotype that included branched dendrites; after 7 days, extended projections were seen (Figures 4(c) and 4(d)). The neural differentiated cells were either bipolar or multipolar. The number of cells showing neuronal morphology was increased when using the two-step method through sphere formation compared to the one-step method of induction of a monolayer culture.

**Figure 5:**
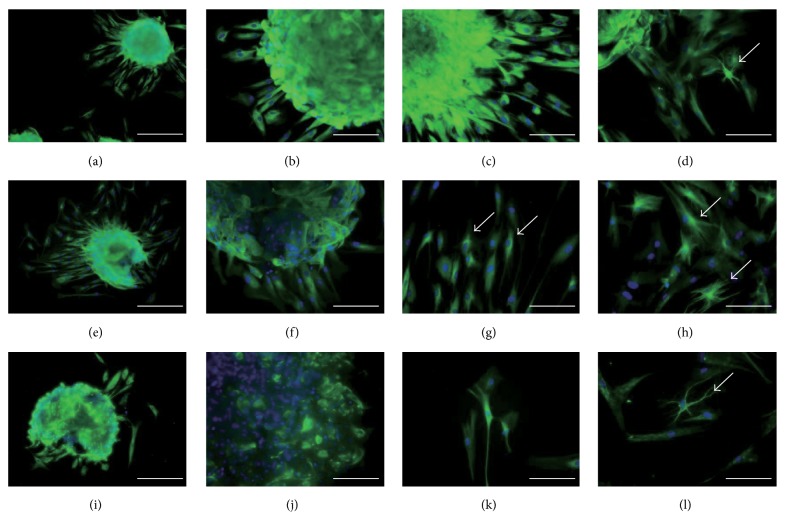
Immunofluorescence staining for neural markers in hDFCs following two-step neuronal induction. (a–d) Nestin staining. (e–h) *β*-III-Tubulin staining. (i–l) S100*β* staining. (a, b, e, f, i, j) A neurosphere cultured in NDM on day 0. (c, g, k) The cells in the neurosphere cultured in NDM on day 3 grew out from the sphere. (d, h, l) The cells in the neurosphere cultured in NDM on day 7 grew out from the sphere. Scale bars (a, e, i) = 100 *μ*m and (b, c, d, f, g, h, j, k, l) = 50 *μ*m. Arrows indicate cells that were strongly positive for the marker and that showed neural-like morphology with branched dendrites and refractile cell bodies.

The cell spheres and the cells that spread out from the sphere were immunoreactive for nestin, *β*-III-tubulin, and S100*β* (Figure 5). Cells with neural morphology were also strongly positive for nestin, *β*-III-tubulin, and S100*β* after culture in NDM for 7 days (Figures 5(d), 5(h), and 5(l)).

### 3.3. Expression of Neural Markers in the Neurosphere

**Figure 6:**
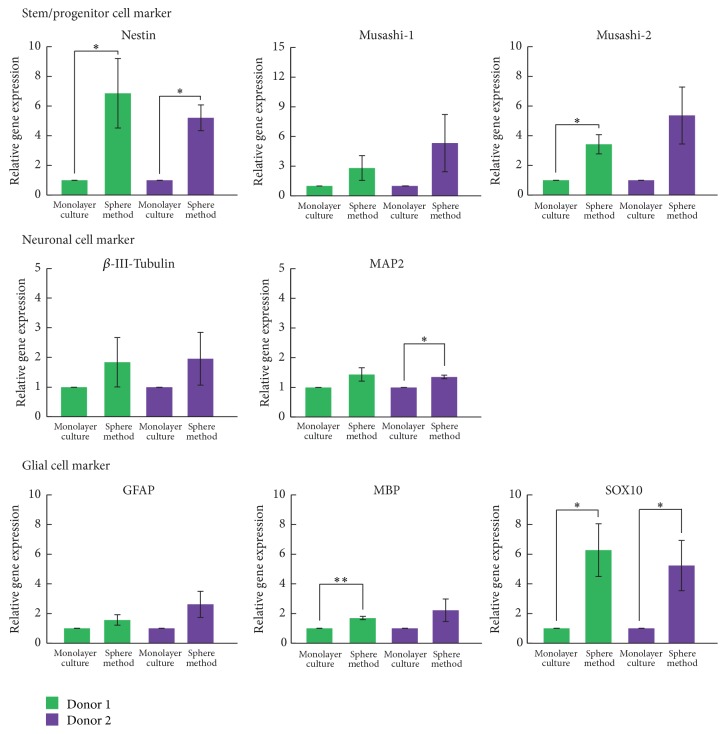
Analysis of gene expression of neural markers after neurosphere formation in hDFCs isolated from two patients. Gene expression was examined in hDFCs in monolayer culture and the neurosphere. Values represent the means ± SD (*n* = 3). ^*∗*^*p* < 0.05,  ^*∗∗*^*p* < 0.01.

To evaluate the efficiency of neurosphere formation for neuronal differentiation, gene expression of neural marker genes was examined with real-time PCR in hDFCs isolated from the two donors. We compared the monolayer culture to the neurosphere culture. The expression of all markers was higher in the neurosphere culture compared to the monolayer culture of hDFCs (Figure 6). Expression of nestin and SRY-related HMG-box 10* (SOX10)* was significantly increased in the neurosphere culture of hDFCs from both donors. Expression of Musashi-2, microtubule-associated protein 2* (MAP2)*, and myelin basic protein* (MBP)* was also significantly higher in the neurosphere culture of hDFCs from only one donor. Expression of other markers was higher in the neurosphere culture, but the difference was not significant.

### 3.4. Expression of Neural Markers during Neuronal Differentiation after Sphere Formation

**Figure 7:**
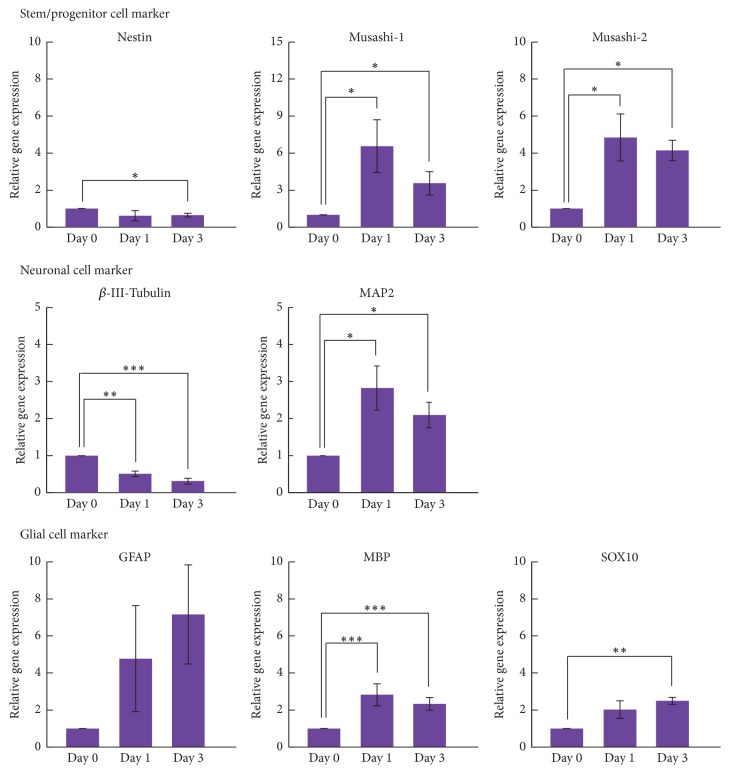
Gene expression of neural markers in hDFCs following two-step neuronal induction. Gene expression was examined in hDFCs during neuronal differentiation after the neurosphere formation. Values represent means ± SD (*n* = 3). ^*∗*^*p* < 0.05, ^*∗∗*^*p* < 0.01,  ^*∗∗∗*^*p* < 0.005.

The gene expression of neural markers was examined in hDFCs during neuronal differentiation after sphere formation. Expression of* nestin* and *β-III*-*tubulin *decreased in hDFCs during neuronal differentiation (Figure 7). Expression of* Musashi-1* and* Musashi-2*,* MAP2*, and* MBP* increased significantly in hDFCs after culturing in NDM, although the expression of these genes was lower in hDFCs after culturing in NDM on day 3 than on day 1 (Figure 7). Expression of glial fibrillary acidic protein* (GFAP)* and* SOX10* increased in hDFCs during neuronal differentiation in a time-dependent manner (Figure 7).

## 4. Discussion

This study was initiated to explore the potential of neuronal differentiation by hDFCs in a monolayer culture on a fibronectin-coated dish and NDM. We observed that the majority of undifferentiated hDFCs expressed nestin, *β*-III-tubulin, and S100*β*, which suggests neurogenic potential. Several hDFCs acquired morphological features of neuronal cells and stained positive for *β*-III-tubulin and S100*β* during neuronal differentiation. Cultured hDFCs displayed heterogeneous phenotypes during neurogenic differentiation. Several studies have shown that MSCs from several tissues such as bone marrow, umbilical cord, and dental pulp cells have the potential for neuronal differentiation. However, only a subpopulation of MSCs differentiates into neuron-like cells* in vitro* because MSCs are a heterogeneous subpopulation.

The technique for expanding neural stem cells in a three-dimensional environment on a nonadherent plastic surface as cell clusters is termed neurosphere culture [21]. Recent studies have shown that MSCs that are seeded on low-attachment plastic tissue culture plates in serum-free medium supplemented with bFGF, EGF, and B27 supplement form floating spheres after several days. During cell sphere culture, hypoxia and the serum-free condition cause mature cells in the intermediate zone to die, but stem cells and neural progenitors survive. Hypoxia not only enhances the stemness of human dental pulp cells but also plays an important role in the maintenance of neural progenitors [22, 23]. In addition, EGF and bFGF treatment enables the cells to better respond to the neuronal differentiation stimuli. Neuron-like cells pretreated with EGF and bFGF stop proliferating, present with longer neurite extensions, and acquire an expression pattern more consistent with a neuronal differentiation program [24].

We induced neuronal differentiation of hDFCs through neurosphere formation to increase the potential of cells that differentiated into neuron-like cells. The number of cells with neuronal morphology was higher using the two-step method through sphere formation compared to the one-step method of monolayer culture. The gene expression of markers for neural cells including neuronal stem cell/progenitors, neuronal cells, and glial cells was examined in the cell spheres compared to monolayer cultures in hDFCs isolated from two patients. The expression of* nestin* and* Musashi-1* and* Musashi-2* was enhanced in hDFCs by sphere formation.* Nestin* expression by pluripotent stem cells is considered to be a prerequisite for the commitment of cells toward the neural lineage [25, 26]. Musashi-1 and Musashi-2 are mammalian neural RNA-binding proteins that are highly enriched in neural precursor cells that are capable of generating both neurons and glia during embryonic and postnatal central nervous system development. Markers of neuronal cells such as *β-III-tubulin* and* MAP2* and glial cell markers such as* GFAP*,* MBP*, and* SOX10* were also increased in cell spheres compared to the monolayer culture in hDFCs. These results suggested that sphere formation induces commitment of hDFCs toward the neural lineage.

We also examined the gene expression of neural markers during neuronal differentiation when the cells were cultured in NDM on fibronectin-coated culture dishes after formation of cell spheres. The expression of* nestin* was decreased during neurogenic differentiation, whereas the expression of* Musashi-1* and* Musashi-2* peaked on day 1. We suggest that the stem cell marker* nestin* may be expressed during the early phase of neuronal differentiation compared to the progenitor marker* Musashi* and that progenitor cells were increased after 1 day of neuronal differentiation. Previous reports have shown that the expression of* nestin* decreases in stem/progenitor cells during neuronal differentiation [18].* Musashi-1* is expressed during early neuronal development of neural stem/progenitor cell cultures from mouse brain and human umbilical cord blood cells. To the best of our knowledge, no comparison analysis has been performed of expression of* nestin* and* Musashi* during neuronal differentiation.

The expression of* MAP2*,* GFAP*,* MBP*, and* SOX10* was upregulated in hDFCs during neuronal differentiation via neurospheres, and the expression of *β-III-tubulin* was downregulated in this study. *β-III-tubulin*, a phosphorylated tubulin that is considered a neural-specific marker, is expressed during the initial stages of brain development [27]. In our study, expression of *β-III-tubulin* was slightly upregulated in porcine neural progenitor cells treated with ciliary neurotrophic factor compared to standard proliferation conditions and then slightly downregulated for 3 days compared to 1 day in ciliary neurotrophic factor.* MAP2*, a neuronal marker, is associated with actin during early axonal development [28]. In contrast,* GFAP*, encoding an intermediate filament protein, and* MBP*, encoding a structural protein in myelin, are markers of glial cells [29].* SOX10* is a transcription factor that is crucial for Schwann cell differentiation.* SOX10* is expressed in neural crest cells and is required for Schwann cell identity and progression beyond the immature stage [30, 31]. Collectively, our results suggest that sphere formation induces commitment of hDFCs toward the neural lineage. Our data revealed evidence of mixed neuronal and glial differentiation of hDFCs when treated with neuronal differentiation conditions. In a previous study, the expression of S100*β* in hDFCs treated with and without neuronal differentiation was induced after birth following SOX10 induction in Schwann cells [32]. Therefore, hDFCs may have potential for differentiation toward the glial lineage rather than the neuronal lineage.

In this study, early neural cell markers were upregulated in hDFCs during neurosphere formation, and late neural markers were upregulated after a second step of differentiation. However, hDFCs are neural precursors without potential for glial cell differentiation because undifferentiated and differentiated hDFCs do not express the glial cell marker GFAP. In our study, glial markers such as GFAP, MBP, and SOX10 were upregulated in hDFCs during neurosphere formation and a second step of differentiation, although we used a different protocol for neuronal differentiation of hDFCs that involved neuronal differentiation medium and a coated dish. In addition, we suggest that the characteristics of hDFCs isolated from individual patients may be different. Although the expression pattern of the neural markers was similar in hDFCs in the two patients, the level of expression was different. Further investigation will be needed to examine the potential for neural differentiation by hDFCs isolated from individual patients and the appropriate cell culture conditions.

This study shows that hDFCs have neural progenitor-like properties and express neural markers in an undifferentiated state. hDFCs acquired neural morphology and upregulated several neural markers in appropriate neural stimulation conditions. In conclusion, we suggest that hDFCs are appropriate candidates for treatment of central and peripheral nervous diseases and injuries due to their regenerative potential and their possible therapeutic role.

## Figures and Tables

**Table 1:** Primer sequences for real-time PCR.

Gene	Forward primer (5′ → 3′)	Reverse primer (5′ → 3′)	Annealing temperature (°C)
Nestin	AACAGCGACGGAGGTCTCTA	TTCTCTTGTCCCGCAGACTT	60°C
*β*-III-Tubulin	AGTGATGAGCATGGCATCGA	AGGCAGTCGCAGTTTTCACA	60°C
Musashi 1	GCCCAAGATGGTGACTCG	ATGGCGTCGTCCACCTTC	60°C
Musashi 2	TTAGGTGATGTCCTCAGACC	GAGAGGGAAACCATCAAGA	60°C
GFAP	GAGGCGGCCAGTTATCAGGA	GTTCTCCTCGCCCTCTAGCA	60°C
MAP2	AGGCGTATGATCTCTTTGAG	GTTTGCTCCTAGGGTTTCTT	55°C
MBP	CTATAAATCGGCTCACAAGG	ATTAGGTAACAGGGGCAAGT	55°C
SOX-10	AGGAGAAGGAGGTTGACTGT	GCATGTCAGACCCTCACTAT	55°C
